# Feasibility of a Center of Mass Based Fuzzy-Logic Phase Detection Algorithm for Post-Spinal Cord Injury Gait

**DOI:** 10.1109/TNSRE.2026.3705681

**Published:** 2026

**Authors:** Gabrielle C. Labrozzi, Musa L. Audu, Nathaniel S. Makowski, Ronald J. Triolo

**Affiliations:** Department of Biomedical Engineering, Case Western Reserve University, Cleveland, OH 44106 USA; Motion Study Laboratory, Louis Stokes Cleveland Department of Veterans Affairs Medical Center, Cleveland, OH 44106 USA; Department of Biomedical Engineering, Case Western Reserve University, Cleveland, OH 44106 USA; Motion Study Laboratory, Louis Stokes Cleveland Department of Veterans Affairs Medical Center, Cleveland, OH 44106 USA; Department of Physical Medicine and Rehabilitation, MetroHealth System and Case Western Reserve University School of Medicine, Cleveland, OH 44109 USA; Motion Study Laboratory, Louis Stokes Cleveland Department of Veterans Affairs Medical Center, Cleveland, OH 44106 USA; Department of Biomedical Engineering, Case Western Reserve University, Cleveland, OH 44106 USA; Motion Study Laboratory, Louis Stokes Cleveland Department of Veterans Affairs Medical Center, Cleveland, OH 44106 USA

**Keywords:** Spinal cord injury, center of mass, fuzzy-logic algorithm, gait

## Abstract

Current approaches for restoring walking post spinal cord injury (SCI) use feedforward systems to apply neuromuscular stimulation to lower extremities to realize stepping. However, abnormal gait patterns still persist. During neurotypical walking, center of mass (CoM) follows well-defined trajectories and reflects whole-body movement that has the potential to be exploited to better control stimulation and improve gait performance. Fuzzy logic algorithms (FLA) mimic human reasoning and provide robust frameworks for noisy inputs typical of information from biological systems. Thus, we examined the feasibility of CoM based FLA to accurately detect four phases of the gait cycle off-line and determine its potential for controlling stimulation to improve walking mechanics post-SCI. Five neurotypical subjects participated in a session of overground walking to develop a CoM based FLA with five inputs that accounted for gait periodicity at various gait speeds. We optimized the FLA with a Genetics Algorithm and completed a cross validation before verifying the system offline with data from one neurotypical person and three individuals with SCI by computing goodness indices (G). The FLA performed well for double support and swing phase (*G* ≤ 0.52) across participants. This study supports the feasibility of utilizing components of the CoM kinematics as features for gait phase detection during walking post-SCI.

## I. Introduction

Spinal cord injury (SCI) can damage central nervous system axons and compromise the descending peripheral nerve activity to the muscles which can result in a reduction or loss of motor function [1]. Walking mobility involves whole-body movement and is important for overall health and independence that is frequently impaired by SCI [2]. Additionally, walking is a top priority of restoration following an SCI with individuals desiring to regain walking function without the usage of assistive devices [3], [4], [5].

To approach these aspirational goals, neuromuscular electrical stimulation (NMES) is a viable option for generating stepping motions or facilitating gait after paralysis [6], [7]. Current systems initiate steps by activating the peripheral motor nerves to contract the paralyzed or paretic muscles in a variety of feedforward paradigms based on neurotypical (NT) electromyography patterns [6] that are heuristically tuned per individual [7]. For instance, a manual finger switch can trigger each step [8], but such feedforward systems require high levels of dexterity and concentration, which limit walking speed [9] and require excessive upper extremity effort (UEE) to maintain upright posture [7].

Automating transitions between phase-specific and individualized feedforward patterns of activation to achieve stepping motions has been accomplished by acquiring inertial measurements (i.e., acceleration signals) from sensors attached to forearm crutches [10] or a single IMU at the hip or mounted on a walker [11]. However, such finite-state systems utilizing transition rules based on simple thresholds on individual accelerometer signals may require extensive subject-specific adjustments to ensure accurate triggering and stepping, which is undesirable because they are time consuming, labor intensive, and nongeneralizable. Furthermore, despite NMES assistance at multiple muscles at appropriate times and intensities, abnormalities can still remain such as observably discontinuous, asymmetrical, and variable gait behaviors and require high metabolic expenditure [7]. We assume that information from a more global control parameter, rather than a series of individual sensor signals, may reduce tuning and capture the totality of gait. Thus, allowing NMES -assisted walking to be more intuitive and natural, and addressing the current limitations of existing systems to improve efficiency of walking and maximize functional outcomes.

The center of mass (CoM) is a global parameter that can be thought of as the point at which the entire mass of the body can be effectively located to describe the kinematics and kinetics of the body as a whole [12]. It can be computed analytically or estimated via motion capture, ground reaction forces, or inertial measurement units [13]. CoM has been applied to human locomotion, especially in motor neuroprostheses and gait rehabilitation as it describes whole-body movement during gait, associates with dynamic stability [14], follows well-defined sinusoidal patterns in NT individuals [12] and changes as a consequence of injury or disease [15], [16], [17]. Previous research has demonstrated the success of CoM surrogates with feedforward control for both nonconsecutive [18] and consecutive stepping [11] in the SCI population. Thus, CoM kinematics are promising and information-rich control parameters for feedback applications and modulating stimulation to facilitate human locomotion after paralysis.

Fuzzy logic algorithms (FLA) are a non-linear, logical, rule-based system approach [19] that, unlike other paradigms, mimics human reasoning and decision-making [20]. The system is composed of four components: knowledge base, fuzzification, decision-making, and defuzzification [21]. Input data are converted into linguistic variables to which the system then applies memberships and rules to determine the necessary output. Due to the internal structure of the membership functions (MF), FLA provide robust frameworks when handling uncertainty, noise, and fluctuations in data input [20]. This is important when considering post-SCI gait where abrupt input changes may occur due to physiological disturbances such as spasticity, excitation-contraction coupling, variable responses and contractile properties, and similar factors. Additionally, FLA assign the inputs a degree on a zero to one scale instead of a binary value [20], and enable expert knowledge to inform the system rather than computing the hidden behavior in data such as with artificial neural networks [22]. FLA have been used successfully to discriminate between gait phases in both NT [23] and pathological [24] walking. While successful at accurate gait phase detection post-SCI, Skelly et al. observed errors of the order of 300ms delays in state transition times [24]. Since typical peripheral nerve stimulation interpulse intervals can approach 50ms [7], reducing such delays is important to avoid missing stimulation dynamics that are necessary to achieve effective forward progression and balance. Furthermore, the controller designed by Skelly and Chizeck had MF that had to be tuned for every individual user [24]. Creating a generalized FLA may accommodate for a degree of intersubject variability and decrease the required time consumption and labor intensity of heuristically tuning transition rules.

The purpose of this study was to investigate the feasibility of the components of the CoM kinematics as features for detecting phases of the gait cycle post-SCI via a FLA. Since an overarching goal with restoring gait after paralysis is to approach NT gait patterns, as it aligns with the aspirational goals of individuals with SCI, we validated our approach on both NT and SCI gait dynamics off-line, with no prior work as a comparable setup. We hypothesized that CoM kinematics have four distinct clusters of features that detect phases of the gait cycle accurately with minimal delay.

## II. Methods

### A. NT Experimental Procedure

**Fig. 1.**
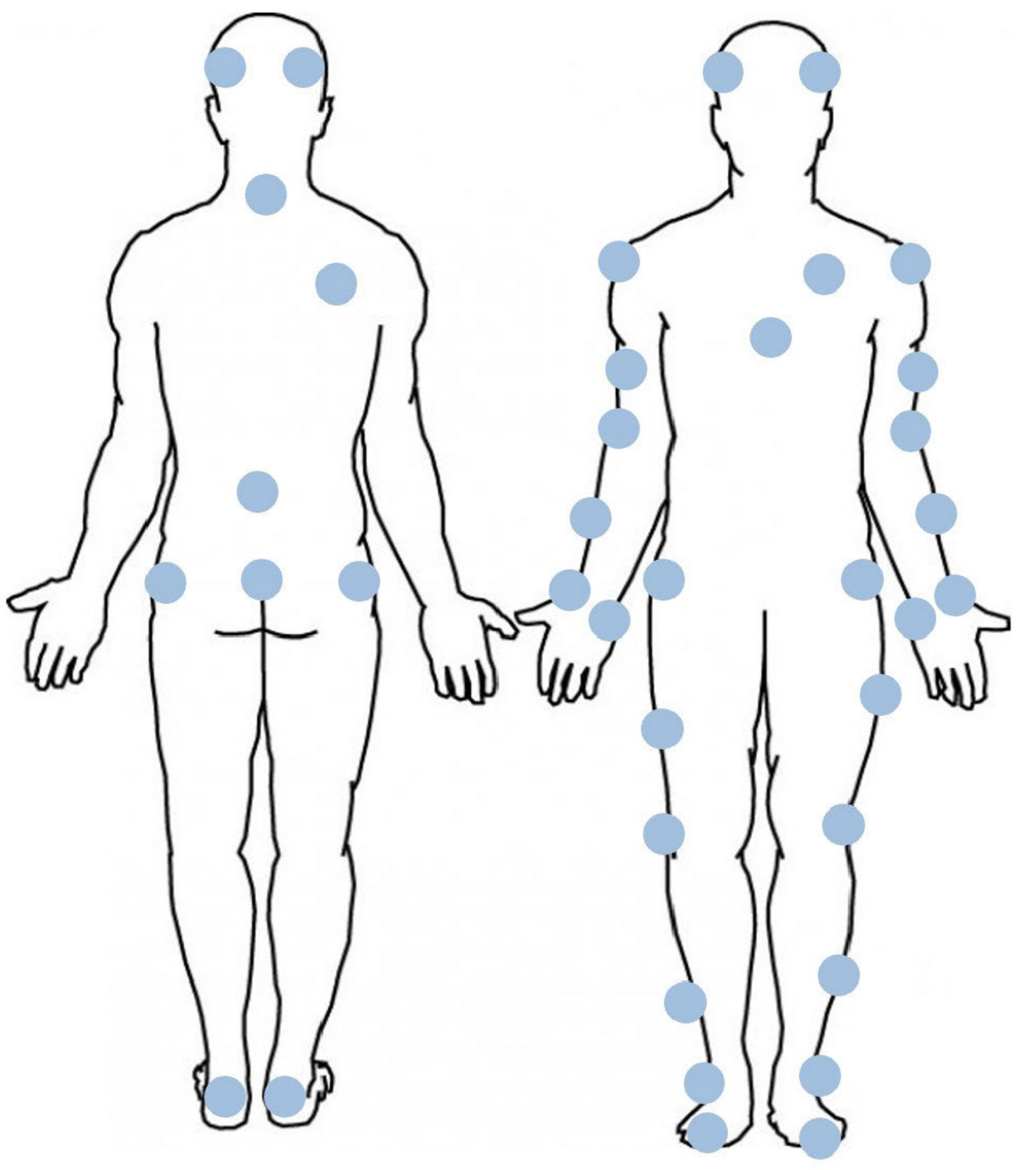
Body mounted sensors. The blue dots are positions of the 38-reflective marker set.

Quantitative gait analysis data collected from five NT individuals were used to discretize CoM kinematics and generate the distributions of each component for the FLA [13]. Anthropometric information on the five NT individuals, labelled NT01 to NT05, are listed in (Table I). We utilized 80-150 strides extracted from walking across the 10m working volume of a 16-camera Vicon motion capture system to identify the profiles of CoM parameters, which included walking at subjectively slow and at comfortable speeds since CoM varies with gait velocity [12]. The data included walking speeds from 0.39m/s to 2.4 m/s where the median walking speed for NT01 was 0.58m/s, NT02 = 1.01m/s, NT03 = 0.85m/s, NT04 = 0.85 m/s, and NT05 = 1.10 m/s. Conditions also included walking continuously with and without a walker to capture data reflecting baseline NT walking as well as emulating pathological patterns with an assistive device. The customized walker had a 67 × 77mm base with a variable height based on participants preference and stature. Three-dimensional CoM position was computed analytically for every sample, at a sampling rate of 60Hz, from a modified Plug-in Gait reflective marker set [25] that comprise of the proximal and distal points of each body segment (Fig. 1) and anthropometric tables [26]. The data were further low-passed filtered at 15Hz, and referred to a reference frame located in the middle of the stance foot of each step offline via the methods of Labrozzi et al. [13]. CoM position components were then differentiated numerically to derive their velocities and accelerations with an applied simple moving average filter with a window of 5 [27] for minimal signal distortion. With a 60Hz sampling rate, this equated to a lowpass filter with 3dB cutoff of 6Hz. We normalized the CoM components for the effects of gravity and height for each participant via Equation 1, and scaled the results for all steps within each trial so each component of *CoM* ∈ [−1, 1].

(1) $$CoM_{\hat{p}}=\frac{CoM_{p}}{l},CoM_{\hat{\nu }}=\frac{CoM_{\nu }}{\sqrt{gl}},CoM_{\hat{a}}=\frac{CoM_{a}}{g}$$

where *CoM_p,v,a_* are CoM position (*p*), velocity (*v*), and acceleration (*a*), ${\mathrm{CoM}}_{\hat{p},\hat{v},\hat{a}}$ are the equivalent normalized values, *l* is the subject’s height, and *g* is the acceleration due to gravity.

### B. Fuzzy Logic Algorithm Development

**Fig. 2.**
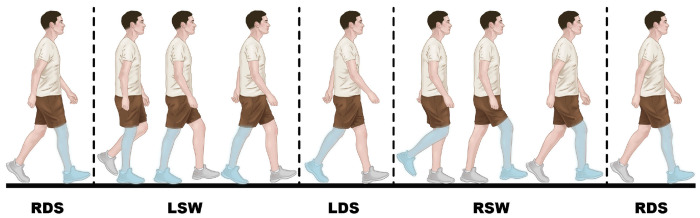
Gait cycle schematic. The gait cycle was divided into four phases: right double support phase, RDS, left swing phase, LSW, left double support phase, LDS, and right swing phase, RSW.

The FLA utilized a Mamdani system with five inputs and one output. To formulate the knowledge base, we separated CoM kinematics per trial into four phases of the walking cycle: right and left double support phase (RDS and LDS), and swing phase (RSW and LSW). We indicated the separation between DS and SW from the inferiosuperior (IS) kinematics of the heel and toe markers off-line. The local minima of the IS position of the toe marker data indicated toe-off, and the instance of zero IS velocity of the heel marker indicated heel contact. These points in time defined the transitions between actual gait phases and were verified by visual inspection. The subphases utilized in this study are defined and illustrated in Fig. 2.

**Fig. 3.**
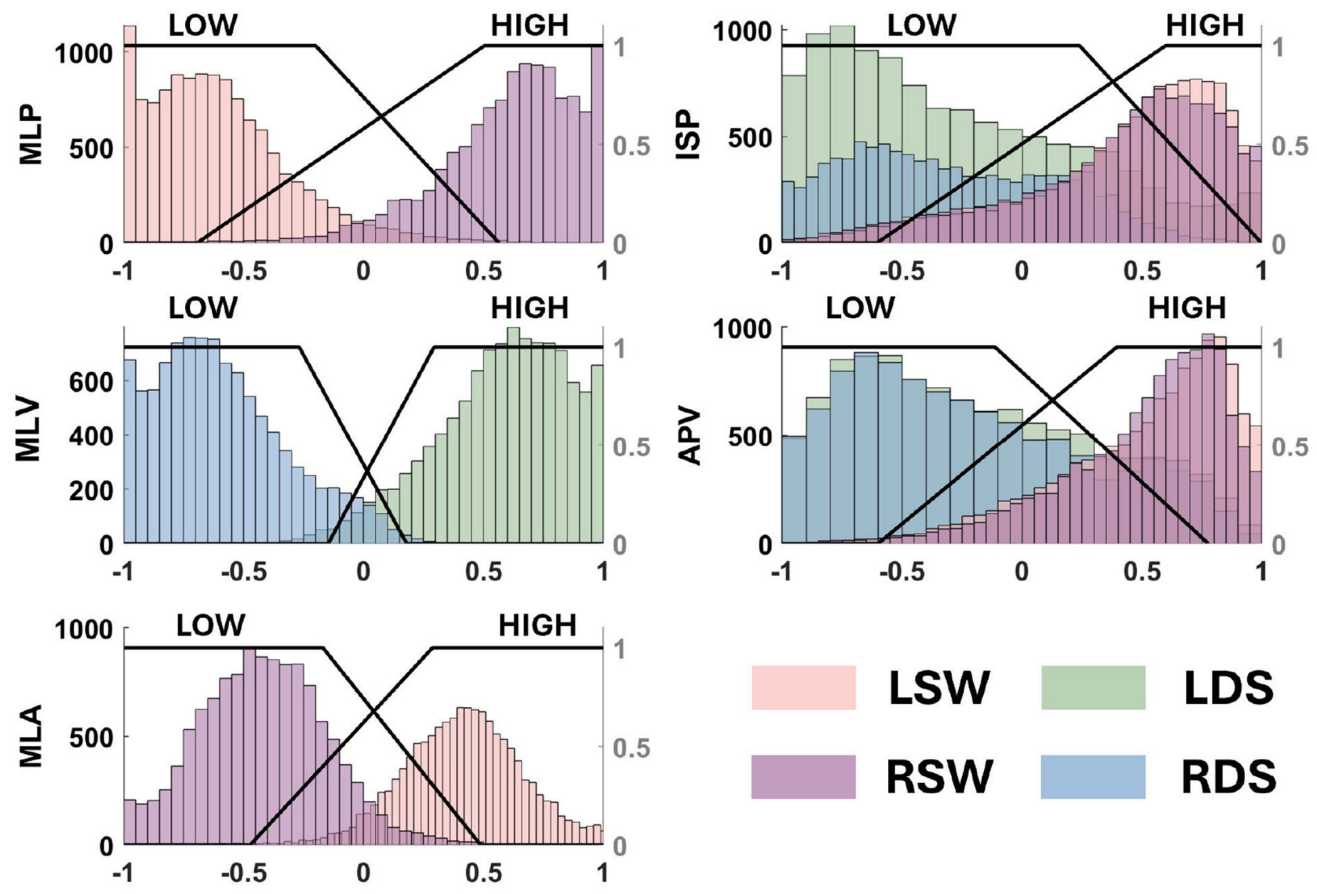
Input membership functions. The CoM distribution normalized between [−1,1]. Bar colors indicate the CoM in a specific gait phase: LSW = Left Swing Phase, LDS = Left Double Support Phase, RSW = Right Swing Phase, RDS = Right Double Support Phase. The left y-axis represents the number of instances a CoM value fell into each bin. The black lines indicate the membership functions per input on a [0, 1] scale (right y-axis). The linguistic variables are either LOW or HIGH.

We generated the histogram plots from four out of the five participant data from the parsed normalized CoM kinematics for the five inputs: CoM mediolateral (ML), anteroposterior (AP), and IS position (*CoM_MLP_*, *CoM_ISP_*,), velocity (*CoM_MLV_*, *CoM_APV_*), and acceleration (*CoM_MLA_*) (Fig. 3). These kinematic parameters were selected based on the separation between DS and SW for the right and left leg. The independent variable is the normalized CoM kinematics between [−1, 1] and the dependent variable is the number of instances where the CoM value fell into a certain bin. To simplify the FLA, each input focused on a selection of gait phases where the distribution modes were the furthest apart compared to the other subphases.

#### 1) Input Membership Functions Optimization:

**Fig. 4.**
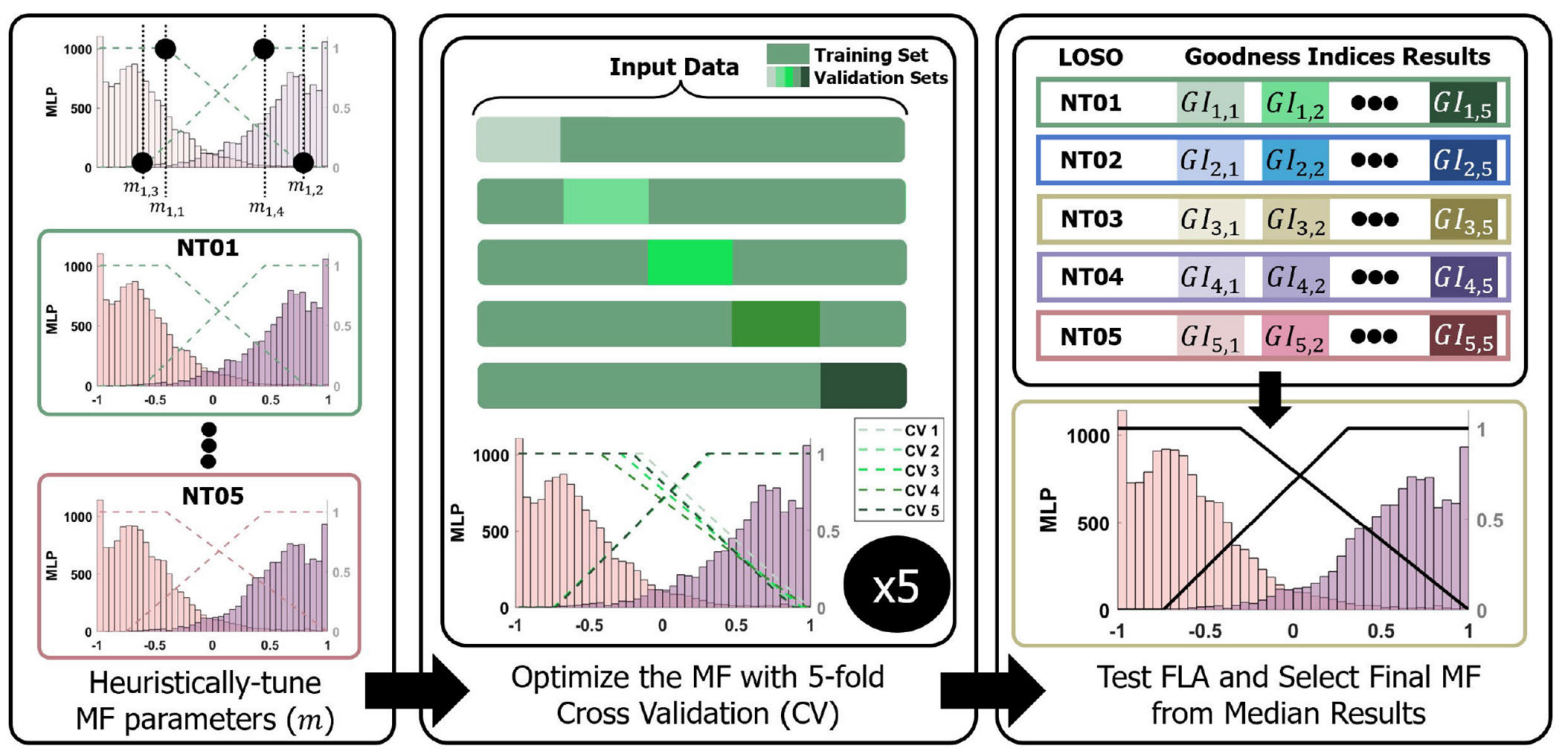
Optimization process. A Leave-One-Subject-Out Cross Validation (LOSO) was completed when optimizing the MF. MF were heuristically defined based on CoM end (*m*_1,2_ and *m*_1,3_) and peak (*m*_1,1_ and *m*_1,4_) values (**LEFT**). An internal k-fold cross validation was performed for each LOSO with a k of five (**MIDDLE**). We selected the final MF based on the median goodness indices (GI) per internal cross validation and across the LOSO groups (**RIGHT**), which was the third LOSO group indicated by the yellow box.

We described the initial input MF using trapezoidal and triangular functions (Fig. 4, **LEFT**) and based on preliminary observation, each function was classified as either LOW or HIGH and illustrated in Fig. 3. For each of the two functions, there are two tunable transition parameters, *m*, including the support and core bound points where the MF change from zero to nonzero and from non-zero to one, respectively (Fig. 4, **LEFT upper pane**). The quantitative location of these parameters were initially heuristically defined. However, for consistency across all inputs, we used an optimization algorithm to determine the optimal locations for the parameters. Once the parameters are specified, they fully define the shapes of the MF, which in turn affect the accuracy of the resulting predicted gait phase outputs.

The optimization cost function, J, was the root mean square error between the predicted (*y′*) FLA and actual (*y*) gait phase output *y′* (*m*), *y* ∈ ℕ|1 ≤*y′*, *y*≤4} where 1 = LSW, 2 = LDS, 3 = RSW, 4 = RDS.

(2) $$J\left(m\right)=\sqrt{\frac{\sum_{i=1}^{N}{w_{GP}}^{*}{\left(\left\|y_{i}-y_{i}^{\prime }\left(m\right)\right\|\right)}^{2}}{N}}$$

where *N* = total number of data points, *i* = sample point, *w_GP_* represents the weight for a given phase where *w_DS_* = 0.40 and *w_S W_* = 0.60, *m* is a [5 × 4] matrix of the MF transition points (x4) for each input (x5).

We imposed parameter constraints like requiring the min/max values of each support point to be within 20% of the initial condition and further bounding the core point by the midline of the LOW/HIGH MF. Each gait phase was weighted to avoid overwhelming the cost function and were defined by their contribution towards an efficient walking cycle. For instance, SW was weighted highest (0.60) because it is necessary for limb advancement and the continual progression of dynamic walking [28], making it the most critical for ensuring a continuous gait pattern that minimizes the effort and reliance required on the upper extremities with assisted devices. Detecting DS was considered the second most important (0.40) since it correlates with the activation of the extensor muscle groups to propel the body forward, ensure stability, and absorb shock [28]. These weighting values were arbitrarily selected as an initial guess and were subject to adjustment per run of the optimizer to help with convergence. The final cost function we used to test the capabilities of our model implemented the following weights: *w_DS_* = 0.45 and *w_SW_* = 0.55.

The optimization problem used a Leave-One-Subject-Out Cross Validation (LOSO). For each iteration, data from four of the five participants were included in the generation of CoM distribution plots (Fig. 4, **LEFT**), while the data from the remaining participant were used to test the predictive capability of the models. For training/validating the model, we performed a k-fold cross validation test with a k of 5 to avoid overfitting (Fig. 4, **MIDDLE**) [29]. Each dataset was separated into a training and validation set with an 80/20% split in the number of sample points.

For solving the optimization problem, we utilized a Genetic Algorithm from the *MATLAB 2023b Global Optimization Toolbox* (The MathWorks, Inc., Natick, MA) since it is appropriate for FLA tuning [30], [31], easily handles constraints, and avoids getting trapped in local minima [32]. We ran the optimizer across multiple repetitions until there was no significant change in the objective function. For each repetition, the previous MF with the median accuracy were used as initial conditions. The final MF included data from four out of five participants from one LOSO group (Fig. 4, **RIGHT**). All final MF are illustrated in Fig. 3. This selection was based on the overall median score in the goodness indices, (G), which is a global index and expressed as [33]:

(3) $$G=sqrt\left({\left(1-\left(\frac{TP}{TP+FN}\right)\right)}^{2}+{\left(1-\left(\frac{TN}{FP+TN}\right)\right)}^{2}\right)$$

where *TP*, *FN*, *TN*, *FP* are the total number of decisions in a set of testing trials where the data for the proposed FLA was classified as a *true positive (TP), false negative (FN), true negative (TN),* or *false positive (FP)* when compared to the same data point for the actual gait phase. At a sampling rate of 60Hz, *G* was based on at least 9,770 individual decisions made on each data point.

From our optimization process, the final MF did not include data from NT03. Therefore, we used this participants data as an additional testing group to verify the accuracy of the developed CoM-based FLA without additional fine-tuning.

#### 2) Output Membership Function and Defuzzification Interface:

**Fig. 5.**
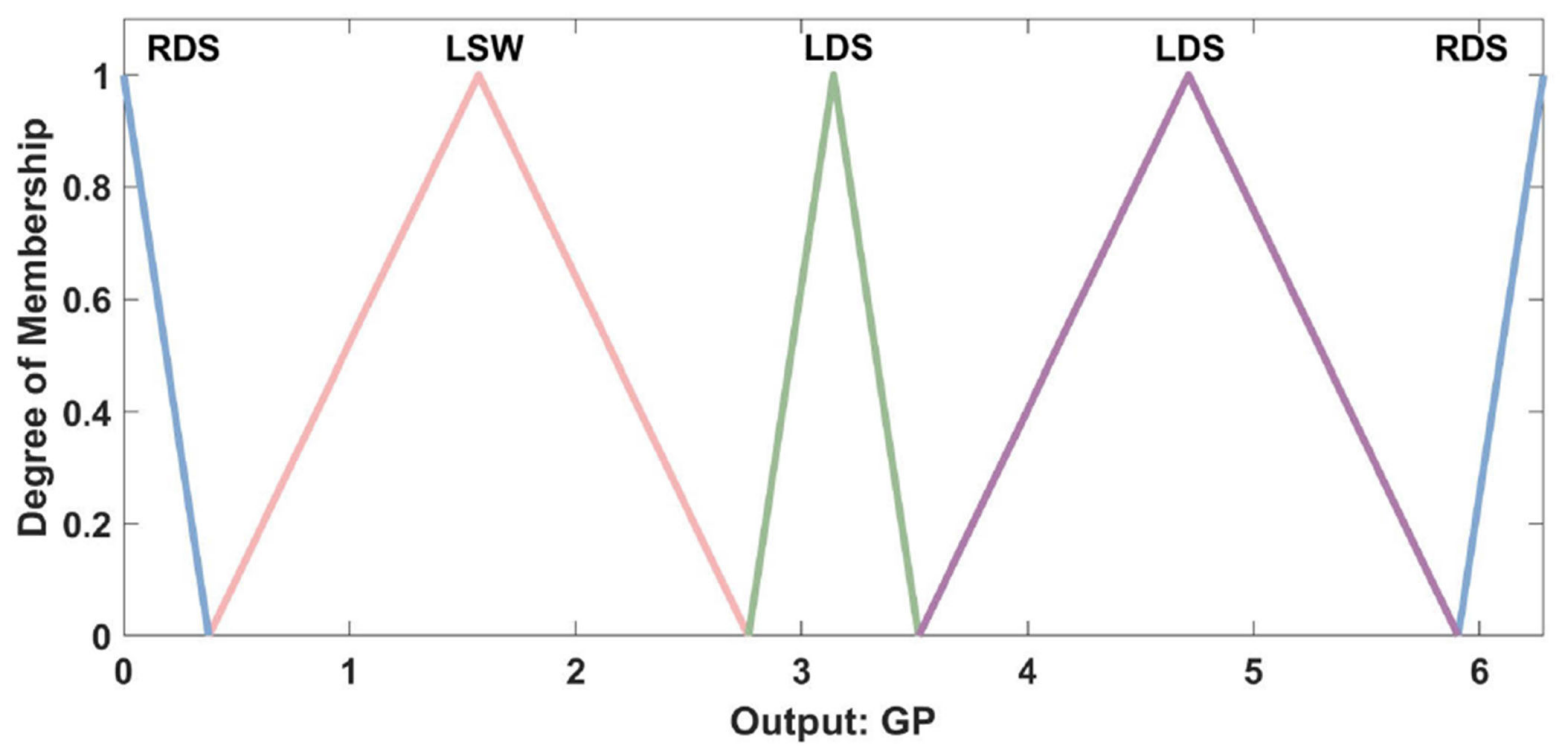
Output membership function. The percentage for each gait phase (GP) varied depending on the gait speed. The example above is for medium speed (>0.60*m/s* and ≤ 1.50*m/s*).

The output MF for the four gait phases is depicted in Fig. 5. The time within each gait phase is dependent on walking speed [34]. For simplicity, we separated SW and DS into three bins: 66/34% split at slow gait speeds (≤ 0.60*m/s*), 72/28% for medium (between 0.60*m/s* and ≤ 1.50*m/s*), and a 76/24% split at fast speeds (≥ 1.50*m/s*). Due to the cyclic nature of walking, we employed a periodic function where the output ranged from [0, 2*π*] and implemented the work of Mitsuishi *et al.,* for the FLA defuzzification interface [35]. Table II defines the transition rules for deciding the gait phase output based on the input linguistic variables, identified in Fig. 3.

#### 3) Supervisory Rule:

A supervisory rule was used to ensure continuous progression through the predicted gait cycle, and guarantee participant safety by avoiding undesirable changes of stimulation with future real-time application to pathological gait. The rule allowed the system to either remain in the previous phase or transition to the next phase of the gait cycle, preventing skipping past gait phases. For example, if the system is in RDS, the rule allows the system to only output RDS or LSW.

### C. Verification With Pathological Gait

We verified the suitability of CoM kinematics as features for detecting gait phases in pathological gait by testing the developed CoM-based FLA, as described above, on data from individuals with incomplete SCI (iSCI) without further fine-tuning. Three individuals with chronic iSCI of varying injury severity, and no uncontrolled spasticity, vestibular compromise, or interfering musculoskeletal issues other than paralysis (labelled SCI01 to SCI03) participated in two walking sessions (Table I) to minimize potential confounds from fatigue. Ambulatory categories were objectively assigned based on experimentally measured gait speed [36]. Participants signed consent forms approved by the Institutional Review Board of the Louis Stokes Cleveland VA Medical Center (protocol 1591730, approved 3/11/2021).

Across the two sessions, participants completed at least 100 steady-state strides without stimulation along the 10-m walkway at their preferred speeds (median speeds: 0.71m/s = SCI01, 0.47m/s = SCI02, and 0.41m/s = SCI03). Each participant wore a 38-reflective marker set (Fig. 1), and used their typical assistive device (Table I). A physical therapist provided contact guarding for safety. We followed the same procedure described above to process the iSCI marker data and compute the five CoM parameters.

### D. Statistical Analysis

Performance of the FLA in predicting gait event transitions was assessed off-line by the goodness index (G), Eq. 3. When interpreting *G*, *G* ≤ 0.25 is optimal, 0.25 < *G* < 0.70 is good, *G* = 0.70 is random, and *G* > 0.70 is bad [33]. Additionally, transition time differences (TTD) were computed to determine if the CoM-based FLA estimated transition led or lagged the actual moment of transition. Due to the implementation of the supervisory rule, the calculation of the TTD ignored when the FLA failed to transition into the subsequent phase and got stuck/suspended for at least one gait cycle. Instead, this scenario was accounted for with the percentage of suspensions in a given phase. The percentage of suspensions was defined as:

(4) $$\%\;Suspensions_{GP}=\frac{\#\;Gait\;Cycles\;FLA\;is\;stuck\;in\;GP}{Total\;\#\;of\;Gait\;Cycles}*100$$

where *GP* is a given gait phase.

A Shapiro-Wilks Test with an *α* = 0.05, for *G* indicated that the majority of results were non-normally distributed [37], so we conducted a Wilcoxon Rank Sum Test with *α* = 0.05, 0.01, 0.001 to statistically compare the median *G* to determine if NT and the SCI data from each of the participants were from continuous distributions with equal medians [38]. The significance levels are indicated accordingly in the results.

## III. Results

Table III contains the Median (Interquartile Range) for the four gait phases for *G*. The FLA performed well for DS and SW (*G* ≤ 0.52) for both NT03 and iSCI data. There was significant improvement in *G* for SCI02 in RSW (p= 0.001) relative to NT03. The accuracy of detection significantly declined for DS and SW for SCI01 and SCI03 when compared to NT03. However, the *G* values were still considered *good*.

Bottom half of Table III highlights the outcome of implementing the supervisory rule with the % suspensions. The CoM-based FLA got minimally stuck in RDS for SCI02 (< 5.9%) yet had the largest % of suspensions for NT03 and SCI01 (21.1 and 27.6% respectively). The CoM-based FLA got stuck for at least once gait cycle for all phases for SCI03 (2.0% RDS, 2.7% LSW, 12.6% LDS, and 0.7% RSW). RSW had the least amount of suspensions across all participants. The algorithm had medium TTDs of −16.7ms and 33.3ms with interquartile ranges of 183ms and 100ms for SW and DS respectively. The TTDs varied across all participants with no discernable pattern.

## IV. Discussion

The CoM-based FLA performed optimally for RDS and LSW on a NT participant data, which was expected since they had greater separation in the *CoM_IS P_* distribution compared to LDS and RSW (Fig. 3). LDS had greater than 200 sample points for each bin across the entire [−1, 1] span while RDS had less than 50 sample points at *CoM_IS P_* values > 0.65. The greater separation between RDS and the swing phases enabled the algorithm to detect RDS and LSW with greater certainty. Our G_SW,DS_ results for NT03 and SCI02 are qualitatively comparable to other off-line gait detection methods that observed G values from 0.12 ≤ G ≤ 0.32 [33].

Surprisingly, there were higher percentages of suspensions for NT03 and SCI01 compared to SCI02 and SCI03. This may stem from CoM dependence on gait speed as observed by Tesio et al. [12]. NT03 and SCI01 walked the fastest at approximately 0.85m/s and 0.71m/s, respectively. As gait speed increases, the variation in the *CoM_MLP,APP_* displacements decrease while *CoM_IS P_* excursions increase. The changes in the displacement variation may have increased the uncertainty of transitioning out of RDS into LSW. However, the number of suspensions for LDS were minimal for NT03 and SCI01. Thus, there may be an underlying asymmetrical factor that contributed to the higher suspensions in RDS that requires further investigation.

The algorithm lagged overall for SW phases compared to DS which was expected. While the modes of the *CoM_IS P_* distributions for DS and SW were separable, approximately −0.7 and 0.7, respectively, the DS tails extended to values of one. Thus, it may take longer for the CoM-based FLA to recognize that the transition into swing has occurred, leading to an overall lag time.

Overall, the algorithm performed well when introduced to pathological gait, and surprisingly performed significantly better for SCI02 when compared to NT03 for *G_RDS_*. As previously stated, CoM is dependent on walking speed [12] with a median gait speed of 0.85m/s for NT03 and 0.47m/s for SCI02. The variation in *CoM_MLP_* displacement increases at lower gait speeds [12], which may explain the greater separation in SCI02s CoM distributions (Supplementary Figure 1, *TOP*) when compared to the NT distributions (Fig. 3). Thus, raising the certainty for decision-making by the CoM-based algorithm. While SCI03 walked at a relatively similar speed to SCI02, 0.41m/s, the algorithms performance for SCI03 declined and was significantly worse compared to NT03, which was expected due to SCI03 having a more severe impairment (Table I). SCI03 presented with observably greater asymmetrical patterns, especially in the AP and ML directions (Supplementary Figure 2, *BOTTOM*), which contributed to signal drift. Thus, the CoM distributions had greater overlap (Supplementary Figure 1, *BOTTOM*), leading to poorer performance in *G*.

Underlying compensatory strategies may have contributed to the algorithms observed performance post-SCI. SCI01 exhibited an inconsistent, wide lateral sway of the trunk to remain stable, which resulted in variable oscillations in *CoM_MLP_* as presented in Supplementary Figure 2, *MIDDLE* between 2.5 to 4.5 seconds. SCI01 did not shift over his right stance limb to the same degree as his left. This led to the algorithm periodic suspensions in various gait phases. SCI03 utilized forearm crutches, which has previously been reported to differ observably from NT gait patterns. In particular, crutch walking impacts certain gait phase durations and timing of events, such as heel strike, significantly compared to NT [39]. Hip rotation has also been shown to increase in crutch gait, impacting the center of gravity [40]. These differences may have factored into the uncertainty in SCI03 CoM distributions and the poorer algorithm performance. We accounted for assistive technology via a walker in the simulated pathologic gait performed by the NT participants since it impacts gait post-SCI [41], but we did not include asymmetry in the FLA formulation due to single or bilateral crutches or canes. However, it is important to emphasize that despite the addition of compensatory strategies and asymmetrical gait in our SCI testing group, the CoM-based algorithm performed well. Thus, our results support the feasibility of the components of the CoM kinematics as features for gait detection after SCI.

### A. Limitations of the Current Study

One limitation of this study is the relatively small testing sample size. While we tested the algorithm on data from three individuals with iSCI with varying injury levels and severities with promising results, the SCI population is highly heterogeneous [42]. Thus, a larger scale trial that represents a wider selection of the SCI population is needed to determine robustness and complete generalizability. Another limitation is the age range of our NT participant pool for the generation of the FLA knowledge base component. This pool is younger compared to the average age of injury [43], and CoM has been shown to differ across NT age groups [44]. However, the impact age has on CoM in the SCI population remains unclear. Although the results for SCI02, the oldest participant, suggest this limitation did not affect our results, further exploration is required to determine the effect of age on the algorithm robustness. Additionally, we tested the FLA on four male participants. There are significant differences in gait kinematics between genders [45], which can be expected to impact CoM [46]. Further research is necessary to fully characterize the effect of gender on algorithm accuracy. Lastly, this study employed motion capture to estimate CoM kinematics off-line to minimize introducing errors while training and testing the CoM-based approach. However, this estimation process is impractical for real-time implementation. Future applications of any CoM-based algorithm would need to implement a method to estimate CoM in real-time, such as that described by the work of Labrozzi et al. which employed wearable inertial measurement units positioned on the thoracic region and lower extremities [13].

## V. Conclusion

This study outlines the framework and verification of a CoM-based algorithm for detecting transitions between four gait phases during NT and iSCI walking via a fuzzy-logic approach. The algorithm performed well on all testing participants without additional fine-tuning, suggesting the potential for generalizability. Furthermore, the accuracy of the results indicate the feasibility of the components of the CoM kinematics as features for gait assistance post-SCI. SW and DS were clearly and distinctly separated based on CoM kinematics, allowing the algorithm to have certainty in its decision-making and performance for SCI participants. A supervisory rule can alleviate anticipated errors in the detection across all phases and ensure a continuous progression of the gait cycle. The CoM-based algorithm handled gait asymmetry, compensatory strategies and various assistive technologies well. Prior to clinical implementation, a larger scale trial that addresses the heterogeneity of the SCI population should be performed to establish the robustness and complete generalizability of the technique. While the internal structure of FLA make it robust to noise and our formulation performed well with asymmetric gait typical of household or limited community ambulators with iSCI, further investigation is required to determine algorithm accuracy when introduced to input error from fluctuations in gait speed, more severe gait impairments, inherent delays of real-time applications, or other potential confounding influences.

## Supplementary Material

supp1-3705681

supp2-3705681

This article has supplementary downloadable material available at https://doi.org/10.1109/TNSRE.2026.3705681, provided by the authors.

## Figures and Tables

**TABLE I:** Participant Information

Subject	Gender	Age (yrs)	Height (cm)	Weight (kg)	Months since Injury	AIS Score	Injury Level	Assistive Device(s)	Ambulatory Category
NT 01	M	37	187.9	69.8	X	X	X	X	X
NT 02	M	26	184.2	76.2	X	X	X	X	X
NT 03	F	24	165.1	55.8	X	X	X	X	X
NT 04	M	24	181.6	70.3	X	X	X	X	X
NT 05	F	26	172.1	62.8	X	X	X	X	X

SCI 01	M	29	182.9	83.9	14	C	L2	Bilateral AFO	Community
SCI 02	M	64	172.7	121.9	10	D	T1	Cane (Right Side)	Limited Community
SCI 03	M	48	190.5	108.9	73	C	C6	Forearm Crutches	Household

AIS = American Spinal Injury Association (ASIA) Impairment Scale, AFO = Ankle Foot Orthoses.

**TABLE II:** Transition Rules

FLA INPUT					Gait Phase
MLP	ISP	MLV	APV	MLA	
NA	LOW	LOW	LOW	NA	RDS
LOW	HIGH	NA	HIGH	HIGH	LSW
NA	LOW	HIGH	LOW	NA	LDS
HIGH	HIGH	NA	HIGH	LOW	RSW

NA indicates when a rule did not consider a specific input

**TABLE III:** Fuzzy-Logic Algorithm Performance

Parameter	Subject	Gait Phase			
		RDS	LSW	LDS	RSW
Goodness Index (u)	NT03	0.24 (0.28)	0.23 (0.24)	0.29 (0.22)	0.29 (0.21)
	SCI01	0.34 (0.17)***	0.45 (0.14)***	0.40 (0.18)**	0.52 (0.25)***
	SCI02	0.21 (0.13)	0.19 (0.11)	0.23 (0.13)	0.14 (0.08)***
	SCI03	0.44 (0.20)***	0.37 (0.22)**	0.37 (0.18)*	0.48 (0.29)***

% Suspensions	NT03	21.1	0.0	1.0	1.0
	SCI01	27.6	10.3	3.4	0.0
	SCI02	5.9	0.0	0.0	0.0
	SCI03	2.0	2.7	12.6	0.7

Values are written as Median (Interquartile Range).

* *p* = 0.05 |

** *p* = 0.01 |

*** *p* = 0.001.

The coloring for each *G* aligns with its interpretation: *green = optimal*, and *yellow = good*.
