# Supplementary material for: Feasibility of a Center of Mass Based Fuzzy-Logic Phase Detection Algorithm for Post-Spinal Cord Injury Gait
Source: IEEE Trans Neural Syst Rehabil Eng. Author manuscript; Available in PMC 2026 Jul 27. (PMC13406588; doi:10.1109/TNSRE.2026.3705681)
Supplement: supp1-3705681 [file NIHMS2192267-supplement-supp1-3705681.docx]

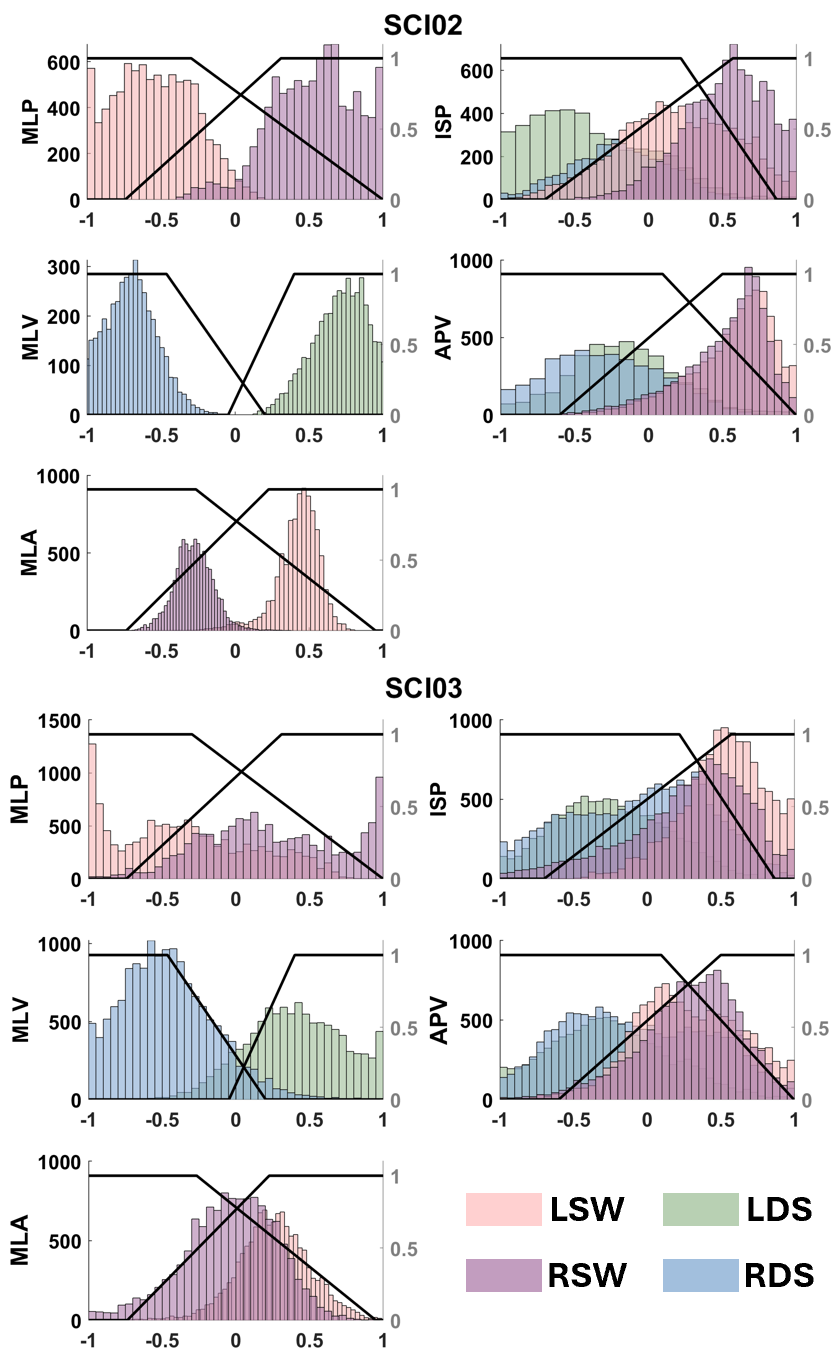


**Supplementary Figure 1: Input membership functions for iSCI.** The CoM distribution normalized between $\left[ -1,1 \right]$ for SCI02 (TOP) and SCI03 (BOTTOM). Bar colors indicate the CoM in a specific gait phase: LSW = left swing, LDS = left double support, RSW = right swing, and RDS = right double support. The black lines indicate the membership functions per input. The linguistic variables are either LOW or HIGH.
