# Supplementary material for: Feasibility of a Center of Mass Based Fuzzy-Logic Phase Detection Algorithm for Post-Spinal Cord Injury Gait
Source: IEEE Trans Neural Syst Rehabil Eng. Author manuscript; Available in PMC 2026 Jul 27. (PMC13406588; doi:10.1109/TNSRE.2026.3705681)
Supplement: supp2-3705681 [file NIHMS2192267-supplement-supp2-3705681.docx]

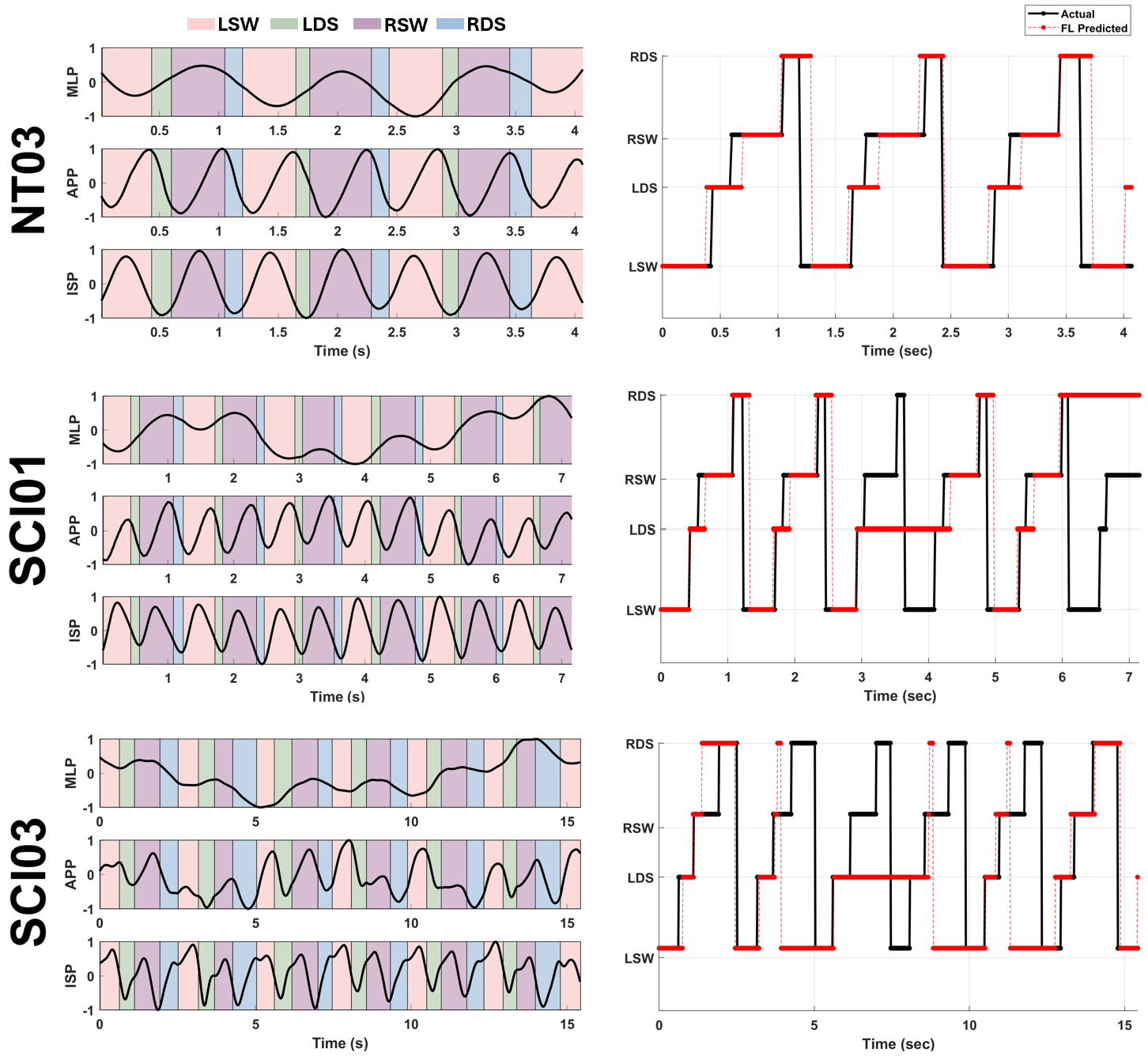


**Supplementary Figure 2: FLA output example**. An example trial for NT03 (TOP), SCI01 (MIDDLE), and SCI03 (BOTTOM). The LEFT plot is the CoM positions for ML, AP, and IS directions (black line) with the breakdown of each gait phase (color bars). The RIGHT plot is the given gait phase for the trial with the actual (black) and CoM-based FLA predicted (red) output.
